# Supplementary material for: Discovery and pharmacophoric characterization of chemokine network inhibitors using phage-display, saturation mutagenesis and computational modelling
Source: Nat Commun. 2023 Sep 16;14:5763. doi: 10.1038/s41467-023-41488-z (PMC10505172; doi:10.1038/s41467-023-41488-z)
Supplement: Supplementary file 4 — Source Data [file 41467_2023_41488_MOESM4_ESM.zip › SourceData/scripts/xngs_20230809084627.tar!/xngs/analysis/SV_EVAA7_logo.pdf]

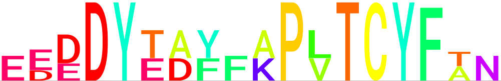

HD534	–	–	–	–	D	D	Y	T	A	Y	–	A	P	L	T	C	Y	F	T	N	S
HD744	–	–	–	–	–	D	Y	E	D	F	F	K	P	V	T	C	Y	F	A	N	S
HD845	–	–	E	D	E	D	Y	E	D	F	F	K	P	V	T	C	Y	F	–	–	–
HD883	–	–	–	–	E	D	Y	E	D	F	F	K	P	V	T	C	Y	F	A	N	–
HD842	–	–	–	E	D	D	Y	T	A	Y	–	A	P	L	T	C	Y	F	T	N	–
HD540	–	–	–	D	E	D	Y	E	D	F	F	K	P	V	T	C	Y	F	A	–	–
HD2134	L	E	E	E	D	D	Y	T	A	Y	–	A	P	L	T	C	Y	–	–	–	–
HD7	–	E	E	E	D	D	Y	T	A	Y	–	A	P	L	T	C	Y	F	–	–	–
HD2	–	–	E	E	D	D	Y	T	A	Y	–	A	P	L	T	C	Y	F	T	–	–
